# Evaluation of Catalytic Effects of Chymotrypsin and Cu^2+^ for Development of UV-Spectroscopic Method for Gelatin-Source Differentiation

**DOI:** 10.1155/2017/2576394

**Published:** 2017-10-08

**Authors:** Anis Hamizah, Ademola Monsur Hammed, Tawakalit Tope Asiyanbi-H, Mohamed Elwathig Saeed Mirghani, Irwandi Jaswir, Nurrulhidayah binti Ahamad Fadzillah

**Affiliations:** ^1^International Institute for Halal Research and Training (INHART), International Islamic University Malaysia (IIUM), Gombak, Kuala Lumpur, Malaysia; ^2^Plant Sciences Department, North Dakota State University, Fargo, ND, USA; ^3^Biotechnology Engineering Department, International Islamic University Malaysia (IIUM), Gombak, Kuala Lumpur, Malaysia

## Abstract

The consumers interest in gelatin authentication is high due to allergic reactions and adoption of Halal and Kosher eating cultures. This research investigated browning development due to enzymatic hydrolysis and presence of Cu^2+^ during Maillard reaction of fish, porcine, and bovine gelatin. The rate of browning index samples showed two phases—rapid and slow—for all the gelatin samples and changes in browning index (Δ*B*_index_) were increased (>100%) in presence of Cu^2+^. Δ*B*_index_ of enzymatic hydrolysates were different among the gelatin species. Fish gelatin hydrolyzate displayed > 400% increase in browning in the first six hours compared to gelatin hydrolyzates from porcine (200%) and bovine (140%). The variation in Δ*B*_index_ of chymotrypsin digested gelatin in presence of Cu^2+^ could be valuable for the development of an efficient UV-spectroscopic method for gelatin differentiation.

## 1. Introduction

Maillard reaction is a nonenzymatic browning leading to formation of numerous compounds when proteins (such as gelatin) are heated in presence of sugar. Maillard reaction products (MRP) have fluorescence and browning capability suitable to measure reaction progress [1]. The degree of browning during Millard reaction depends on the type of sugar, temperature, pH, reaction time, concentration of reactants, presence of inorganic compounds, and most especially protein amino acids profiles [1–3]. The colour intensity of MRP from basic amino acids was reportedly greater than that of acidic amino acids, while nonpolar amino acids were of intermediate colour intensity. Also, browning was accelerated by the presence of metal ions (Fe^2+^ and Cu^2+^) but not affected by Na^+^ [3]. Study of Maillard reaction of essential amino acids with glucose revealed that amino acids were degraded differently and exhibited a varying browning degree [2].

Previous studies have shown that the amino acid profiles and film properties of different gelatin sources varied, most especially methionine and histidine [4]. The content of imino acids proline and hydroxyproline of fish collagen is generally lower than that of mammalian collagen [5]. Another report showed that properties of gelatin film depend on the source of the gelatin such that fish gelatin exhibited lowest water vapor permeability while pork gelatin exhibited least water solubility [6]. Therefore, there is a possibility that variation in the degree of browning in Millard reaction of different gelatin sources could be observed. The degree of browning can be detected by UV-spectroscopy and the readings can then be used to differentiate gelatin of varying sources.

We presume that distribution of aromatic amino acid on gelatin is species specific. Chymotrypsin cleaves peptide bond by aromatic residues, unlike trypsin that only cleaves peptide bond at lysine and arginine. Therefore, chymotrypsin digest of gelatin will possibly produce peptides and hydrolysates unique to different gelatin species. Previous studies have reported enzyme hydrolysis of gelatin yield peptides as a biomarker for gelatin-source differentiation [7]. Most of these studies made use of expensive and difficult procedures such as GC-MS, PCR, ELISA, and HPLC. This research is based on two principles: (i) the chymotrypsin hydrolysis of gelatin from a different source could produce peptides of varying properties that will yield a different degree of browning during Maillard reaction and (ii) the introduction of Cu^2+^ will expedite the rate of reaction and reduce the time for brown colour development.

Till date, there is only one study on the use of UV-spectroscopy reading of the degree of browning of MRP of gelatin for species-specific differentiation purpose. Bovine and porcine gelatin were successfully differentiated with UV-spectroscopy reading after ribose-induced Maillard reaction [8]. A literature search revealed a lack of studies on Maillard reaction of gelatin hydrolyzate and the effect of Cu^2+^ on the degree of browning of gelatin. In a preliminary study, Cu^2+^ has shown to improve browning index during Maillard reaction. Improvement of UV-spectroscopic measurement for differentiation of gelatin power could be achieved by considering effects of chymotrypsin and Cu^2+^ on the degree of browning after Maillard reaction process.

Hence, the aim of this study is to investigate the development of brown colour during Maillard reaction of fish, porcine, and bovine gelatin as affected by chymotrypsin hydrolysis and the presence of Cu^2+^. It is hoped that detail understanding of reaction kinetics will be valuable towards the development of more efficient and reliable UV-spectroscopy based method for species-specific gelatin-source differentiation.

## 2. Methodology

### 2.1. Production of Gelatin Hydrolyzates

Gelatin hydrolysates were produced using chymotrypsin to digest gelatin from fish, porcine, and bovine. The digestion was carried out for 4 h at 25°C with the enzyme-gelatin ratio of 1 : 250 (w : w). The reaction was stopped by heating the mixture at 100°C for 10 min. The solutions were centrifuged at 3000 rpm for 15 min and the supernatants were discounted off and referred to as gelatin hydrolyzates solution.

### 2.2. Nonenzymatic Browning of Gelatin and Gelatin Hydrolysate

An equal volume of 0.25% xylose solutions and 1.0% of gelatin/hydrolyzates were thoroughly mixed to make a final solution containing 0.125 and 0.5% of xylose and gelatin/hydrolyzates, respectively. In another set of experiments, about 2 mM of CuCl_2_ was added to the mixture. The nonenzymatic browning was carried out by heating the mixture at 95°C for 6, 12, and 24 h. The mixture was allowed to cool to room temperature before the determination of browning index.

### 2.3. Determination of Browning Index

The browning index (*B*_index_) of a cooled mixture of gelatin/hydrolyzate containing xylose with or without Cu^2+^ was measured at 420 nm using a microplate spectrophotometer. The change in the browning index (Δ*B*_index_) was used to determine the effect of enzyme hydrolysis and presence of Cu2+. *B*_index_ and Δ*B*_index_ were determined using

(1) Bindex  of  sample=Ax-g−Ag,ΔBindex  of  xylose-hydrolyzate=Ax-h−Ax-g×100Ax-g,ΔBindex  of  xylose-gelatin  in  presence  of  Cu2+=Ax-gCu−Ax-g×100Ax-g,ΔBindex  of  xylose-hydrolyzate  in  presence  of  Cu2+=Ax-hCu−Ax-g×100Ax-g,

where *A*_g_ is the absorbance of gelatin; *A*_x-g_ is the absorbance of xylose-gelatin mixture; *A*_x-h_ is the absorbance of xylose-hydrolyzate; *A*_x-gCu_ is the absorbance of xylose-gelatin in presence of Cu^2+^, and *A*_x-hCu_ is the absorbance of xylose-hydrolyzate in presence of Cu^2+^.

### 2.4. Data Analysis

All data were collected in triplicate and their average and standard deviations were calculated. Diagrammatic representations were used to present the result for easy understanding.

## 3. Results and Discussion

### 3.1. Nonenzymatic Browning of Fish, Porcine, and Bovine

**Figure 1:**
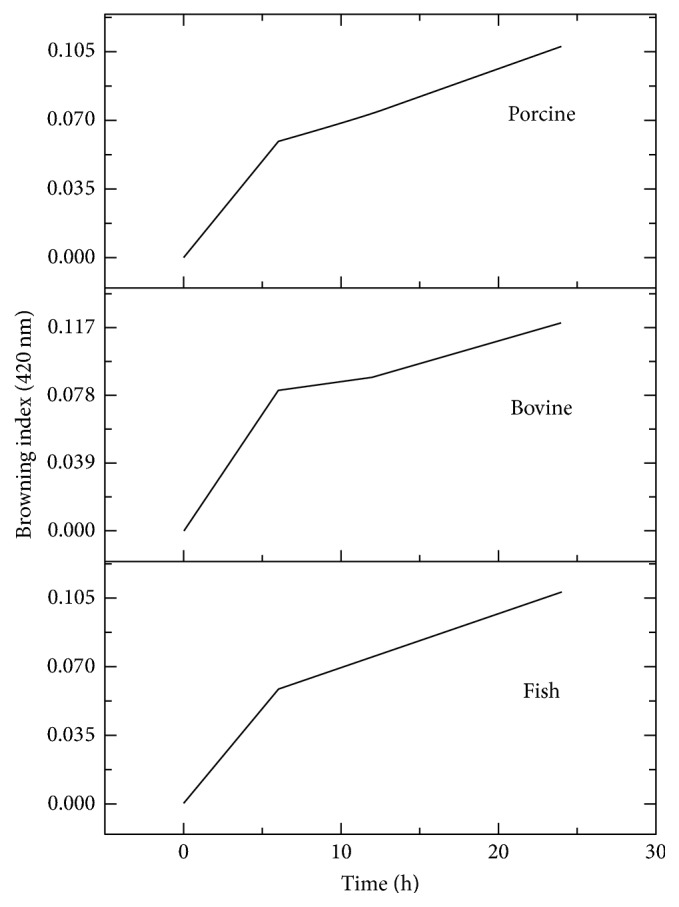
Browning index of xylose-induced nonbrowning of gelatin from different sources (fish, bovine, and porcine) at 95°C.

Figure 1 shows that the browning index of gelatin from different samples increases with increase in heating time. There was an initial rise in the browning index for all the 3 gelatin samples in the first 6 hours followed by a steady increase. This is similar to previous reports that colour formation during Maillard reactions is usually rapid at the early reaction stage [9].

Several studies have shown that the amino acids compositions of gelatin vary according to the species [5]. It is possible that different regions/parts of the gelatin responded differently during the nonenzymatic browning. The variation in the reaction rate might be due to differences in the reactivity of amino acids present in the peptides. A study of Maillard reaction of essential amino acids with glucose revealed that amino acids were degraded differently and exhibited a varying degree of browning [2]. Compared to acidic amino acids, the basic amino acids contributed to browning intensity, while the nonpolar amino acids exhibited intermediate colour intensity [3]. Lysine participated in browning reaction induced by glucose compared to other amino acids and that threonine contributed very little towards browning [2].

This pattern can be explained that there are two regions in the gelatin polymer including the fast responding region and the slow responding region. The slow responding region might need to undergo initial reactions as a sequel to browning reaction. This surmised that gelatin of different sources has similar configurations which eventually reflect similarities in their Maillard reaction kinetic. However, the proportion of the two (slow and quick) regions might vary among the gelatin samples.

### 3.2. Effect of Chymotrypsin Hydrolysis of Gelatin on Degree of Xylose-Induced Nonenzymatic Browning

Structurally, collagen—the parent material of gelatin—is composed of nearly one-third of glycine and another 15 to 30% of proline and 4-hydroxyprolyl. 3-Hydroxyprolyl and 5-hydroxylysyl residues are also present in a smaller amount. Proline and hydroxyproline are responsible for the unique secondary structure of collagen as they limit rotation of the polypeptide backbone and thus contribute to the stability of triple helix [10]. Stabilization of collagen structure involves hydrogen bond (between glycine of the N terminal and proline of the adjacent chain), hydrophobic interaction, and Van der Walls interaction [11]. The collagen intermolar forces are likely inherited by gelatin, therefore, affecting the chemical properties of gelatin during Maillard reaction. The presence of intermolecular forces in gelatin is likely responsible for a reduction on the accessibility of amino acids to xylose during Maillard reaction. Hydrolysis of gelatin might result into peptides that are more reactive and accessible during Maillard reaction.

**Figure 2:**
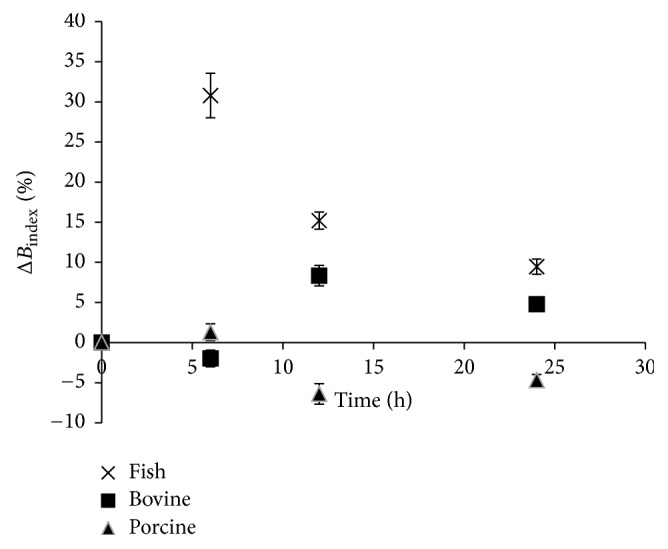
Change in the browning index due of chymotrypsin digestion on xylose-induced nonenzymatic browning of gelatin from different sources. Δ*B*_index_: change in browning index.

In order to determine the effect of chymotrypsin digest of gelatin on contribution to browning index, the percentage difference in browning index of hydrolysates compared to their respective gelatin was estimated and referred to as a change in the browning index (Δ*B*_index_). According to Figure 2, the effect of chymotrypsin digestion on Δ*B*_index_ varies among the gelatin samples. Δ*B*_index_ of fish gelatin hydrolysate was the highest among all the samples throughout the heating period. This suggests that digestion of fish gelatin produces peptides that contributed to Δ*B*_index_ compared to other samples.

Previous reports have shown that enzymatic digestion of gelatin produced different peptides fractions among species. The peptides might contribute differently towards the development of browning during Maillard reaction process and, hence, caused variation of Δ*B*_index_ among the samples.

The trends in browning development that follow were in 2 reaction phases, namely, increasing and decreasing phases. The increasing phase is early and high in fish hydrolysate compared to that of porcine and bovine. The increasing phase of bovine phase occurred only between 6 and 12 h. After 6 h reaction time, there was a decrease in browning in fish hydrolysate while the decreasing phase of porcine hydrolysate occurred after 12 h.

The reduction effect of chymotrypsin hydrolysis on Δ*B*_index_ observed in all the 3 samples might be explained by the lack of stability of their Maillard's reaction products. This reduction in browning occurred during the first six hours of heating in bovine gelatin hydrolysate, while that of fish hydrolysate occurred after the first six hours. Also, the rise in Δ*B*_index_ observed in all samples suggests that initial configuration of gelatin structure hindered the progress of Maillard reaction. Enzymatic hydrolysis did not only increase the surface area of gelatin and expose the amino acid but also reduce the hindering effect from gelatin configuration during Maillard reaction. In line with the findings of Su et al. [12], the smaller molecular peptides obtained from peanut protein exhibited higher reaction degree during Maillard reaction.

As stated earlier, the kinetic of nonenzymatic browning of gelatin of the three samples is similar and comprises fast and slow responding regions. However, enzymatic hydrolysis of gelatin resulted in hydrolysates that varied among the species. The rise in Δ*B*_index_ of fish hydrolysates might be because chymotrypsin hydrolysed the slow reactive regions of fish gelatin to produce more reactive peptides, compared to other gelatin samples. Another reason might be that chymotrypsin hydrolysed bovine gelatin might have produced peptides that participated in Maillard reaction after 6 h.

### 3.3. Catalytic Effect of Cu^2+^ on Degree of Xylose-Induced Nonenzymatic Browning of Gelatin from Different Sources

The presence of Cu^2+^ during the nonenzymatic browning of gelatin samples causes an increase in the rate of more than 100% of *B*_index_. The effect of Cu^2+^ on Δ*B*_index_ of the three gelatin samples was similar such that Δ*B*_index_ increased drastically until the 12 h of heating and then stabilized for bovine and porcine, while that of fish slightly decreased to about 100%. It is possible that Cu^2+^ enhanced formation of MRP and results in loss of slow reaction phase earlier observed in Figure 1. Also, Cu^2+^ might catalyse the formation of MRP from the majority of amino acids and reduced hindering effect of gelatin configuration during the reaction process. This observation agreed with the previous report that stated that Cu^2+^ contributed to the colour intensity of Millard reaction product [3]. The transition metals have been reported to have catalysed Maillard reaction by the oxidative pathway [3]. The presence of transition ions promotes the formation of chromophores and fluorophores during Maillard reaction of DNA with d-Fructose 6-Phosphate [13]. The observed slight reduction in Δ*B*_index_ of fish might be due to loss of stability of MRP after extended heating.

### 3.4. Catalytic Effect of Cu^2+^ on Degree of Xylose-Induced Nonenzymatic Browning of Hydrolysates of Gelatin from Different Sources

The combined effect of enzymatic hydrolysis and Cu^2+^ on the development of browning in Maillard reaction of the gelatin samples is shown in Figure 4. Δ*B*_index_ of fish hydrolysate was highest with a value of 400% followed by that of porcine (200%) and then bovine (140%). In the first 6 h, Δ*B*_index_ increased drastically in all samples and decreased steadily in porcine and fish while that of bovine kept increasing slightly.

**Figure 3:**
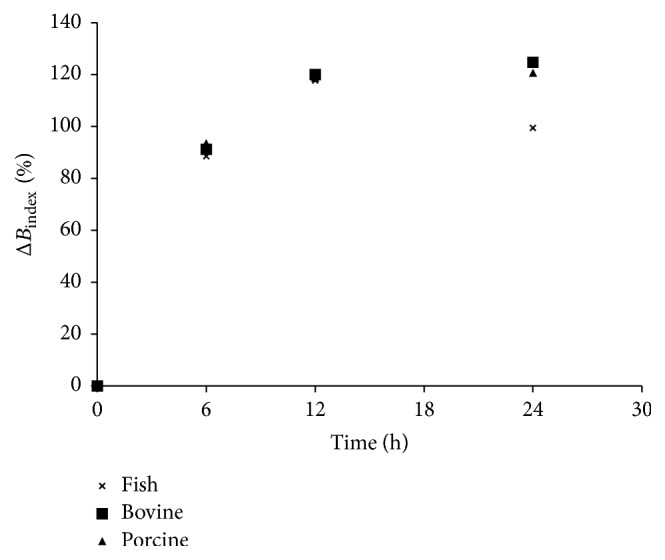
Change in the browning index due to the catalytic effect of Cu ion on the degree of xylose-induced nonenzymatic browning of gelatin from different sources. Δ*B*_index_: change in browning index.

**Figure 4:**
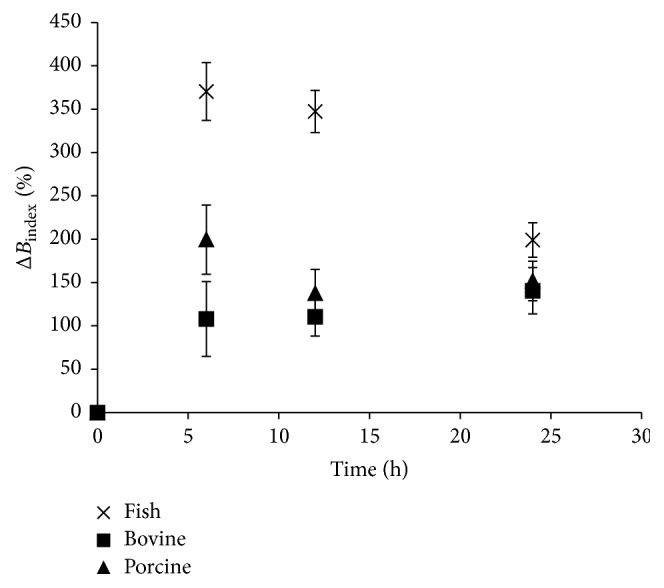
Change in the browning index due to combined catalytic effect of Cu^2+^ and chymotrypsin digestion on the degree of Maillard reaction of gelatin from different sources. Δ*B*_index_: change in browning index.

Compared to that of the result in Figure 3, Δ*B*_index_ was increased in all samples. This suggests that Cu^2+^ catalysed the development of browning of peptides compared to gelatin. At this final stage, differentiating between gelatin from a different source can be achieved by comparing Δ*B*_index_ at different stages. In the early stage, fish gelatin exhibited highest Δ*B*_index_ followed by that of porcine and then bovine. The sharp reduction in Δ*B*_index_ observed in fish gelatin can be used to discriminate fish gelatin from mammalian gelatin.

## 4. Conclusion

Maillard reaction of gelatin from different sources exhibited similar reaction phases—the slow and the fast phases. Enzymatic degradation of gelatin prior to Maillard reaction caused a difference in the production of browning products among the species. Fish gelatin hydrolysate displayed multifold increase in browning in the first six hours compared to gelatin hydrolysates from porcine and bovine. Although the catalytic effect of Cu^2+^ during Maillard reaction was relatively similar in all the gelatin samples, Cu^2+^ affects gelatin hydrolysates differently. The variation in Δ*B*_index_ of chymotrypsin digested gelatin in presence of Cu^2+^ could be valuable for the development of an efficient UV-spectroscopy method for gelatin differentiation. Future works will investigate the effects of other reaction conditions on Δ*B*_index_ of enzymatic hydrolysates from gelatin.
